# Comparative Analysis of Gut Bacterial Diversity in Wild and Domestic Yaks on the Qinghai–Tibetan Plateau

**DOI:** 10.3390/ani14162380

**Published:** 2024-08-16

**Authors:** Tariq Shah, Xusheng Guo, Yongwu Song, Yonggui Fang, Luming Ding

**Affiliations:** 1 Sichuan Provincial Forest and Grassland Key Laboratory of Alpine Grassland Conservation and Utilization of Tibetan Plateau, Institute of Qinghai–Tibetan Plateau, College of Grassland Resources, Southwest Minzu University, Chengdu 610041, China; shah2017@lzu.edu.cn; 2 College of Pastoral Agriculture Science and Technology, Lanzhou University, Lanzhou 730000, China; 3 Probiotics and Biological Feed Research Centre, School of Life Sciences, Lanzhou University, Lanzhou 730000, China; 4 Animal Husbandry and Veterinary Station, Gangcha County, Haibei 812399, China; 5 Key Laboratory of Adaptation and Evolution of Plateau Biota, Northwest Institute of Plateau Biology, Chinese Academy of Sciences, Xining 810008, China

**Keywords:** 16S rRNA sequencing, ruminants, bacterial diversity, grazing yak, gut microbiota

## Abstract

**Simple Summary:**

Comparative analysis of the gut microbiota in wild grazing (WG) and domestic grazing (DG) yaks reveals distinct differences in bacterial diversity, with WG animals exhibiting a higher diversity than DG animals. Firmicutes dominate both groups, with a greater abundance in the WG type, indicating a stronger fiber-degrading capacity. WG yaks have a higher abundance of *Ruminococcaceae* and *Rikenellaceae* families, which are known for their role in fiber degradation, and genus-level differences show a greater presence of fiber-degrading microbes, such as *Ruminococcus* and *Rikenella*. In contrast, DG yaks have a higher abundance of *Prevotellaceae*, *Alloprevotella*, and *Succinivibrio*, associated with protein and carbohydrate degradation, reflecting their different dietary habits. These differences in gut microbiota composition suggest that feeding patterns are crucial in shaping the microbial community, influencing yak health and environmental adaptation. The findings presented herein have significant implications for livestock production, highlighting the importance of considering the impact of grazing practices on gut microbiota, and providing valuable insights for developing prebiotics and microbiological agents tailored to specific dietary needs.

**Abstract:**

The gut microbiota is a diverse and complex population, and it has a key role in the host’s health and adaptability to the environment. The present study investigated the fecal bacterial community of wild grazing (WG) and domestic grazing (DG) yaks on natural grazing pastures, analyzing the gut microbiota using 16S rRNA sequencing to assess bacterial diversity. A total of 48 yak fecal samples were selected from two different grazing habitats. The DG group had more crude proteins and non-fiber carbohydrates. The WG group had more OM, insoluble dietary fiber such as NDF, ADF, ether extract, and TC. There were 165 and 142 unique operational taxonomic units (OTUs) in the WG and DG groups, respectively. Shannon index analysis revealed a higher bacterial diversity in the WG group than in the DG group. At the phylum level, Firmicutes were the dominant bacterial taxa in both groups. The relative abundance of Firmicutes in the WG group was higher than in the DG group. At the family level, the WG group had a significantly higher abundance of *Ruminococcaceae* (*p* < 0.001) and *Rikenellaceae* (*p* < 0.001) than the DG group. The abundances of *Alloprevotella* and *Succinivibrio* were more pronounced in the DG group than in the WG group at the genus level. This study presents a novel understanding of the bacterial communities of ruminants and their potential applications for livestock production.

## 1. Introduction

The Qinghai–Tibetan Plateau (QTP) of China is a vast territory where yaks, cattle, and sheep are the dominant ruminant livestock distributed in a high altitude of 3000 to 5000 m, which covers a region of 2,500,000 km^2^ [1]. The yak (*Bos grunniens*) lives in the high altitudes of the QTP where it has been well-adapted to the harsh environmental conditions (e.g., low temperature, low oxygen, strong ultraviolet radiations, and limited poor-quality forage) [2]. Yaks are generously reproducing and living animals in such a crucial environment of the plateau, which was domesticated by nomadic people more than 7300 years ago [3]. Yaks are the only domesticated animals among all the other livestock that can face food shortages and the harsh environment of the QTP [4]. Yaks, an ancient species of bovine, hold significant importance in the lives of local herdsmen and also occupy a vital ecological niche in the plateau’s environment [5]. Yaks provide people living on the plateau with essential resources like meat, milk, fuel, and warm fur [6]. Yak milk contains caseins, which are antihypertensive agents [7]. Yak milk is rich in healthy fats, like linoleic acid, and essential minerals, like phosphorus and calcium. In Tibet, Nepal, and parts of Mongolia, people consume yak milk regularly as a significant part of their diet [8]. Adjacent to productions such as milk and meat, yaks are also used for local herds’ transportation. Therefore, yaks’ productions are of significance in the economy of local people, owing to its uses and being a source of revenue [9,10]. 

The environment of the QTP is very harsh, mostly in the cold season, because the temperature falls from −5 to −15 °C. During these months (from November to late May) of long snowfall and very cold weather, lesser-quantity and bad-quality grass causes malnutrition and yaks’ retarded growth [11]. Yaks tolerate the harsh conditions with low oxygen because they are endothermic animals. There is no formulated feed provided; although, these yaks are semi-domesticated grazing animals [12]. Early-life malnutrition can disrupt the normal development and functioning of the gut, resulting in growth abnormalities [12,13]. Using various feed additives to enhance the gut health of growth-retarded yaks is crucial for optimal nutrient absorption and normal growth [14]. Research shows that gut bacteria play a crucial role in the health and productivity of farm animals, which is important for agriculture and the economy [15]. The gastrointestinal tract bacterial community plays an important role in the growth and production routine of the animals [16]. The specific bacterial community of the gastrointestinal tract is important for health maintenance and the internal environment of the gastrointestinal tract [15]. 

The rumen is considered a fermentation tank for anaerobic microbial populations, maintaining the internal environment of their host [17]. The rumen microbial community contains bacteria, fungi, and methanogenic archaea, which produce xylanase and cellulases for the fermentation of complex polysaccharides to generate acetate, butyrate, propionate, and volatile fatty acids (VFAs), which are a major source of energy for the ruminants [18]. There is a symbiotic relationship between the host and the microbial community in the rumen. The host provides nutrients and optimum temperature and moisture for the microbes’ best growth, while microbes synthesize protein and digestive by-products such as VFAs [19], which are considered a major source of energy for the ruminants [20].

Microbial composition and diversity in the rumen of ruminants are not only important for good health and production but also for reducing methane (CH_4_) emission [21]. Several factors, including age, species, and season, influence the gut bacteria in animals, but diet has the biggest impact [22]. These factors directly or indirectly influence the rumen microbial community, which might change the physiological response of the ruminants [23,24,25]. The microbial community composition in the rumen is dependent on diet and feeding patterns [26].

Moreover, the rumen microbial variations in yaks are dependent on outdoor and indoor grazing patterns [27]. Major bacterial phyla of yak rumens were recorded as Bacteroidetes and Firmicutes, accounting for (~80%), while low abundances of Fibrobacter, Spirochaeta, and Proteobacteria were (<10%) of the total reads [28]. Although, the dominance of major phyla of Bacteroidetes and Firmicutes are diverse for different yaks [28,29]. Under grazing conditions, some studies have shown that 23 phyla, having 159 families in yak rumen fluid, in which Firmicutes account for 46%, are dominant over Bacteroidetes, which have a 40% presence [29], while another study shows Bacteroidetes being dominant over Firmicutes with 52% and 34%, respectively [28]. 

Research has shown that what yaks eat and where they live (at high or low altitudes) can change the balance of gut bacteria [13,30], but more study is needed to understand how these factors affect their gut health. In particular, few studies have explored how environmental changes affect forage quality and gut microbes in wild and domesticated yaks.Therefore, we used next-generation sequencing to investigate and compare the gut microbiomes of wild and domestic yaks that graze on the same land to identify key differences. We hypothesized that the evolutionary variation of yaks according to climatic changes and pasture forage induces adaptive alterations in the fecal microbial composition. This longitudinal study to evaluate environmental changes in gut microbiota even offers information that could help to improve yak productivity and safety.

## 2. Materials and Methods

### 2.1. The Experimental Site, Animals, and Management

This animal study was reviewed and approved by the Ethics Committee of the College of Ecology, Lanzhou University, Lanzhou, Gansu, China. 

A total of 48 samples were collected from wild grazing and domestic grazing yak groups with different feeding styles and geographical locations (climatic changes) during October 2017. Fecal samples were collected from two groups with different altitudes in Datong County (elevation, 3200 m) and Haixi prefecture (2994 m), respectively, in Qinghai Province, and we used 16S rRNA sequencing on 24 wild grazing yaks in Datong County (WG) and 24 domestic grazing yaks in Haixi prefecture (DG). After the yaks defecated, we used clean cotton swabs to collect feces from the outside and inside of the droppings. We then placed the swabs in sterile tubes, froze them in liquid nitrogen, and stored them at a very low temperature (−80 °C) for further analysis in the laboratory. The WG yak group was randomly selected from a herd with unrestricted access to natural alpine pasture for 24 h a day, while the DG group was selected from a herd of 200 animals that only grazed in the natural alpine pasture from 7 a.m. to 6 p.m. in the daytime. Both groups had free access to water. 

Forage collection was also performed in the same month and year, October 2017, from two geographical locations in Qinghai Province: Datong County (elevation: 3200 m) and Haixi prefecture (elevation: 2994 m). Two groups of yaks were selected, wild grazing (WG) and domestic grazing (DG), with 24 individuals in each group. Forage samples were collected from these areas, respectively, representing their diet.

For the WG group, forage collection was performed in the natural alpine pasture where the yaks grazed freely for 24 h a day. For the DG group, forage collection was performed in the natural alpine pasture where the yaks grazed from 7 a.m. to 6 p.m. The pastures in the area for the DG group were dominated by herbage species of *Kobresia humilis*, *Elymus nutan*, *Kobresia pygmaea*, *Anaphalis lacteal*, *Polyginum viviparum*, *Potentilla fruticose*, *Cortaderia jubata*, and *Sibiraea angustata*, while the pastures in the area for the WG group were dominated by herbage (sedge and grass) species of *Kobresia humilis*, *K. pygmaea*, *K. graminifolia*, *Elymus nutan*, *Polygonum viviparum*, *Anaphalis lacteal,* and also some shrub species, with *Potentilla fruticos*, *Sibiraea angustata,* and *C. jubata* as the dominant non-herbaceous vegetation types.

### 2.2. Determination of Nutritional Composition of Experimental Forages

At 65 °C for 72 h, pasture herbages were dried in an oven, minced, and then passed through a sieve of 1 mm. The measurement of dry matter (DM) was completed by (AOAC, 930.15) at 135 °C for 3 h. The Kjeldahl method (AOAC, 984.13) was used to determine nitrogen using an automatic steam distillation unit, specifically the Kjeltec 2300 Analyzer, manufactured by FOSS, a renowned company based in Hillerød, Denmark. Titration was performed using 0.1 M hydrochloric acid and 1% boric acid. Photometrically, we determined the endpoint. We used nitrogen content multiplied by 6.25 (CP = N × 6.25) to find the crude protein (CP) value. The levels of neutral detergent fiber (NDF) and acid detergent fiber (ADF) were analyzed following the procedure outlined by Goering and Van Soest (1970) [31]. In the NDF method, sodium sulfite was used and values were confirmed for ash contents. Ash content calculation was completed through AOAC, 942.05 [32].

### 2.3. DNA Extraction, PCR Amplification, and MiSeq Sequencing of 16S rRNA Gene Amplicons

A total of 48 fecal samples were thoroughly mixed, and then DNA was extracted from a small amount of each sample (about 0.2–0.3 mg) using a special buffer and a machine that shakes the mixture (called a bead beater). DNA extraction from fecal samples was performed using the QIAamp DNA Stool Mini Kit (Qiagen, Hilden, Germany). The quality and quantity of the extracted DNA were then assessed using the NanoDrop 2000 spectrophotometer (Thermo Fisher Scientific, Waltham, MA, USA). The DNA samples were then adjusted to a concentration of 80 ng/μL before being amplified by PCR. The universal primers 338F (5′-ACTCCTACGGGAGGCAGCAG-3′) and 806R (5′-GGACTACHVGGGTWTCTAAT-3′) were used to amplify the V3-V4 hypervariable region of the 16S rRNA gene in bacterial DNA [33]. The PCR amplification protocol consisted of an initial denaturation at 94 °C for 90 s, followed by 30 cycles of denaturation at 94 °C for 40 s, annealing at 56 °C for 60 s, and extension at 56 °C for 60 s, with a final extension at 72 °C for 10 min. The PCR products were then gel-purified using the GeneJET Gel Recovery Kit (Thermo Scientific, Waltham, MA, USA) according to the manufacturer’s instructions. The purified amplicons were subsequently used for library construction and sequenced on an Illumina MiSeq system using the MiSeq Reagent Kit v2 (2 × 250 bp, Illumina, San Diego, CA, USA).

### 2.4. Sequencing and Data Processing

The QIIME (Quantitative Insights into Microbial Ecology) version 1.7.0 program was used for the organized raw sequence analysis [34]. The barcodes, primer sequences, and low-quality sequences were reduced after sequencing [35]. To obtain efficient tags, the obtained chimera sequences were removed from the dataset using the UCHIME Algorithm with reference to the Gold database [36]. After the process of removing singletons and performing quality control, the SILVA database was used to combine optimized sequence reads, and Release128 http://www.arb-silva.de (accessed on 1 August 2024) was used to cluster effective tags into OTUs with 97% sequence similarity [37,38]. Through representative sequence analysis, the Greengenes database was used for the identification of bacterial taxa [39]. By using QIIME software Version 1.9.1, http://qiime.org/ (accessed on 1 August 2024), Alpha indices such as Chao1, ACE, Simpson, Shannon, and Good’s coverage of bacterial diversity were identified for different treatment groups from whole OTUs table. Meanwhile, to assess the whole structural changes of fecal bacterial communities, the β-diversity of bacteria in the WG and DG groups was visualized using non-metric multidimensional scaling (NMDS) analysis based on ANOSIM and the Bray–Curtis dissimilarity matrix in vegan and ggplot2 packages of R4.1.2. GraphPad Prism version 8.00 for Windows https://www.graphpad.com/ (accessed on 1 August 2024) software was used for the correlation heat map. A co-occurrence network analysis was constructed based on significant genera (*p* < 0.05) with abundance > 0.001 and R-value> 0.4. The co-occurrence network was visualized in Gephi. “Venn Diagram” in R was used for identification of unique and shared OTUs between the WG and DG groups.

### 2.5. Statistical Analysis

Student’s *t*-test in SPSS 16.0 software (SPSS Inc., Chicago, IL, USA) was used to compare the nutrient content of natural pasture grasses. The Wilcoxon rank-sum test with false discovery rate (FDR) correction was employed to identify significant differences in alpha diversity and relative abundances of bacterial phyla, families, and genera. Principal Component Analysis (PCA) was performed using R studio, and the anosim function from the vegan package was used to investigate group-wise differences. Significance was set at *p* < 0.05, and *p*-values were adjusted using FDR to minimize false positives.

## 3. Results

The chemical composition of forages in the domestic grazing (DG) and wild grazing (WG) pastures is shown in Table 1. While the dry matter, organic matter, ash, and hemicellulose content were similar in both groups, the DG pasture had significantly higher (*p* < 0.05) crude protein and non-fiber carbohydrate content compared to the WG pasture. In contrast, the WG pasture had significantly higher (*p* < 0.05) levels of organic matter, insoluble dietary fiber (NDF and ADF), ether extract, and total carbohydrates (TC) compared to the DG pasture.

### 3.1. Analysis of Sequencing Data and Bacterial Diversity

**Figure 1:**
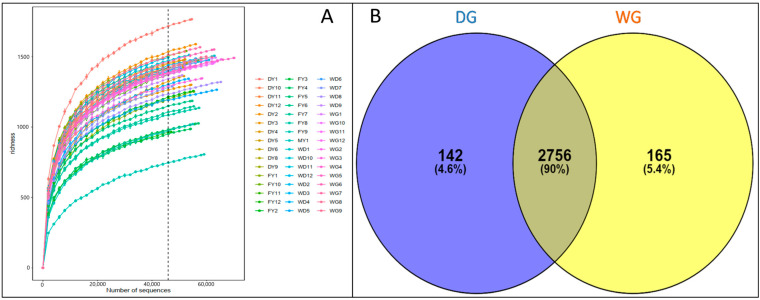
(**A**) The rarefaction curves at 96.8% similarity level of the index of different samples. (**B**) Venn diagram showing operational taxonomic units shared between the two experimental groups, WG and DG.

In total, 4,002,704 raw reads were generated from bacterial 16S rRNA sequencing of 48 samples. After filtering, quality control, and chimera removal, 3,747,261 sequences were obtained with a mean length of 413 bp. The rarefaction curve plateaued, indicating that the number of operational taxonomic units (OTUs) had stabilized and was no longer increasing with additional sequencing data, suggesting that the current dataset was comprehensive and sufficient for analysis. The Good’s coverage of samples was 96.8%, suggesting that maximum bacterial diversity in the samples was recovered (Figure 1A). The OTU analysis revealed 2756 shared OTUs between the two groups, while 165 OTUs were exclusively found in the wild grazing (WG) group, and 142 OTUs were exclusively found in the domestic grazing (DG) group (Figure 1B).

### 3.2. Alpha Diversity

**Figure 2:**
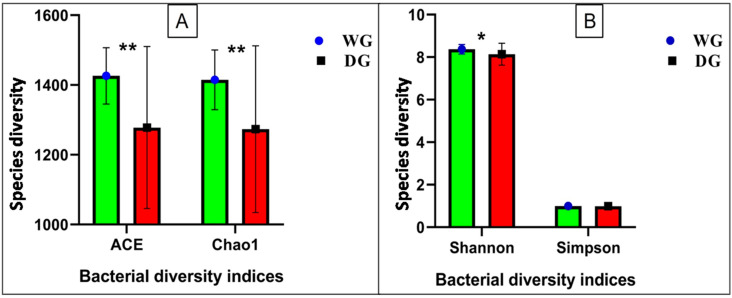
(**A**) ACE and Chao1 indices of bacterial diversity. (**B**) Shannon and Simpson indices of bacterial diversity of WG and DG yak groups. Simpson indices did not show a significant difference between groups (*p* = 0.69). Chao1 and ACE indices were significantly lower in the DG group compared to the WG group, *p* < 0.001= **, *p* < 0.01= *.

The alpha diversity was evaluated using four metrics: the Abundance-based Coverage Estimator (ACE), the Chao1 index, the Shannon index, and the Simpson index. The WG group showed significantly higher (*p* < 0.005) ACE and Chao1 index values (1426.11 ± 80.86 and 1414.86 ± 85.60, respectively) compared to the DG group (1277.99 ± 232.44 and 1273.22 ± 238.97, respectively). The Shannon index (8.37 ± 0.22) was higher (*p* < 0.04) in the WG group than for the DG group (8.13 ± 0.51), whereas the Simpson index did not differ (*p* < 0.69) between the WG (0.99 ± 0.00) and the DG (0.99 ± 0.00) groups, as presented in (Figure 2A,B).

### 3.3. Beta Diversity

**Figure 3:**
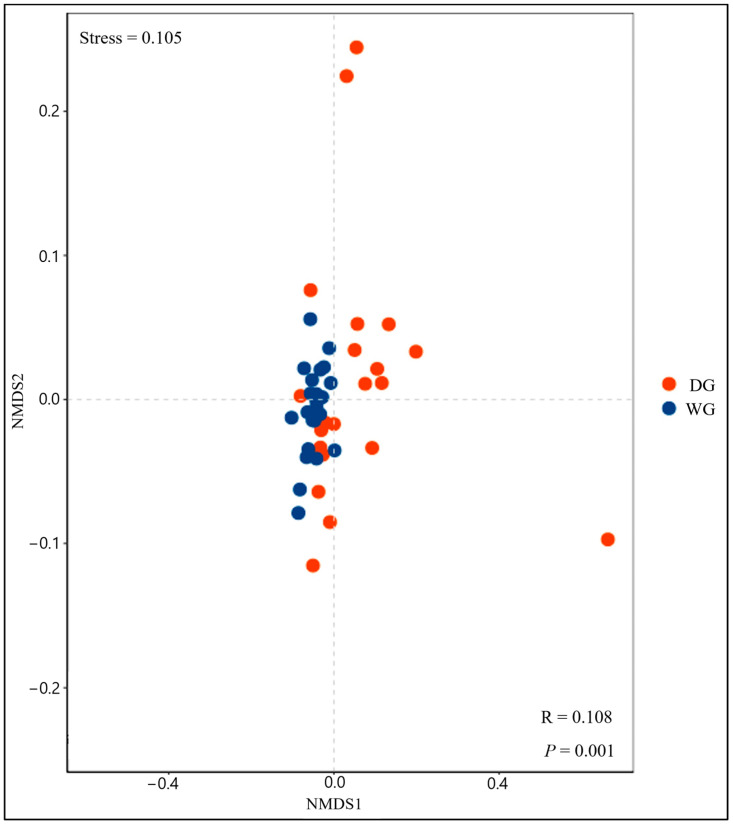
Non-metric multidimensional scaling (NMDS) of bacterial communities at OTUs level; a dot represents each sample, and different colors represent different yak groups, WG and DG.

Non-metric multidimensional scaling (NMDS) analysis revealed that the WG and DG groups formed distinct bacterial clusters in the ordination space (Figure 3), with significant differences at the taxonomic level (*p* < 0.001). However, the bacterial communities of the DG group were more scattered as compared to the WG group, indicating the dissimilarity of the taxonomy between the two groups.

### 3.4. Gut Bacterial Composition

**Figure 4:**
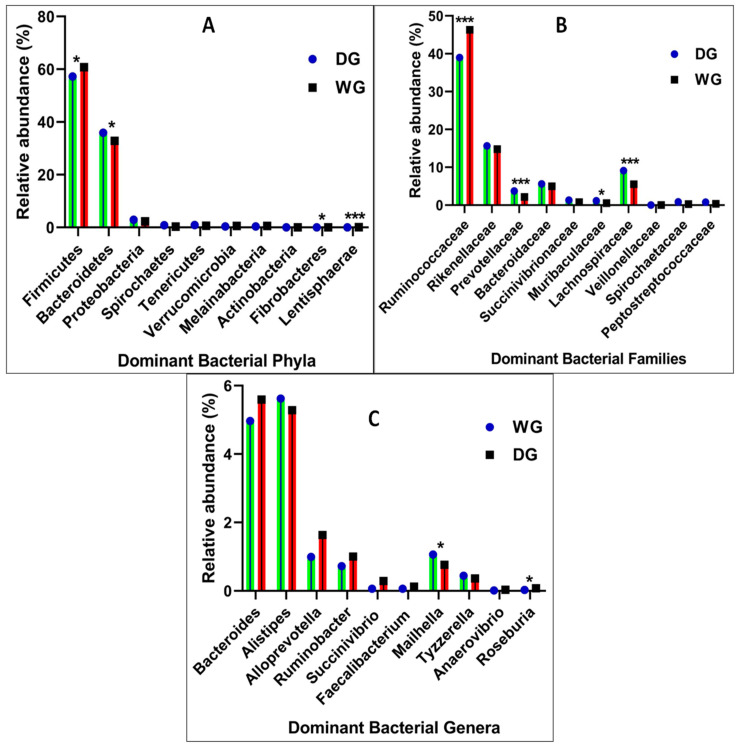
(**A**) Major bacterial phyla found in WG and DG yak groups. (**B**) Major bacterial families found in the WG and DG yak groups. (**C**) Major bacterial genera found in WG and DG yak groups, *p* < 0.0001= ***, *p* < 0.01= *.

The taxonomic analysis showed that the yak gut bacterial community comprised 24 phyla and 228 genera in the groups. The dominant bacterial phyla in the DG group were Firmicutes (57.20% ± 0.05%), Bacteroidetes (35.90% ± 0.05%), and Proteobacteria (2.91% ± 0.02%), whereas, in the WG group, the dominant phyla were Firmicutes (60.76% ± 0.04%), Bacteroidetes (32.80% ± 0.04%), and Proteobacteria (2.31% ± 0.03%) (Figure 4A). Firmicutes in the WG group were significantly higher (*p* < 0.02) than in the DG group, but Bacteroidetes in the DG group were significantly higher (*p* < 0.05) than in the WG group, while no significant variations were recorded for Proteobacteria between the WG and DG groups. Elusimicrobia, Fusobacteria, Kiritimatiellaeota, and Fibrobacteres were less abundant phyla, and their relative abundance in the WG group was significantly higher (*p* < 0.05) than the DG group. The relative abundance of gut bacterial communities at the phylum level in the WG and DG groups is presented in (Table 2).

### 3.5. Relative Abundance of Bacterial Families

At the family level, 129 families were identified in both the DG and WG yak groups. The major and dominant families in the DG and WG groups are presented in (Figure 4B). The most abundant families in the DG group were *Ruminococcaceae* (38.98% ± 0.07), *Rikenellaceae* (15.63% ± 0.04), *Lachnospiraceae* (9.11% ± 0.03), *Bacteroidaceae* (5.59% ± 0.01), *Prevotellaceae* (3.74% ± 0.01), and *Succinivibrionaceae* (1.32% ± 0.02), which accounted for (74.3%) of the total microbial population. *Ruminococcaceae* (46.29% ± 0.04), *Rikenellaceae* (14.78% ± 0.02), *Lachnospiraceae* (5.51% ± 0.01), *Bacteroidaceae* (4.97% ± 0.01), *Prevotellaceae* (2.16%), and *Succinivibrionaceae* (0.79% ± 0.03) were identified in the WG group, accounting for (74.5%) of the total fecal microbiota. *Ruminococcaceae* were significantly higher (*p* < 0.001) in the WG group than in the DG group, whereas *Prevotellaceae* and *Muribaculaceae* were significantly higher (*p* < 0.05) in the DG group than in the WG group. *Bacteroidaceae* and *Rikenellaceae* did not differ (*p* > 0.05) between groups. Some other families were also detected, but their abundance was quite low. The relative abundance of gut bacterial communities at the family level in the WG and DG groups are presented in (Table 3).

### 3.6. Relative Abundance of Bacterial Genera

To further explore the microbial abundance, classification was performed at genus level. The most dominant genus in the DG group was *Bacteroides* (5.59% ± 0.01), with *Alistipes* (5.28% ± 0.01), *Alloprevotella* (1.63% ± 0.01), and *Ruminobacter* (1% ± 0.02) accounting for (13.5% ± 0.01) of all genera (Figure 4C), while the dominant genera of the WG group were *Alistipes* (5.62% ± 0.01), *Bacteroides* (4.97% ± 0.01), *Alloprevotella* (0.99%), and *Ruminobacter* (0.72% ± 0.03), which accounted for (12.3% ± 0.01). Other dominant genera of the DG and WG groups included: *Succinivibrio* (0.29% ± 0.005) and (0.06%), *Faecalibacterium* (0.12% ± 0.002) and (0.06%), *Mailhella* (0.76% ± 0.004) and (1.06% ± 0.003), and *Tyzzerella* (0.36% ± 0.001) and (0.44% ± 0.001), respectively. Variations in the relative abundance of the top 20 genera in two groups, the DG and the WG, were examined. The abundance of *Alloprevotella* and *Succinivibrio* was not significantly (*p* < 0.06) higher in the DG group compared to the WG group. However, the abundance of *Mailhella* was significantly (*p* < 0.01) higher in the WG group compared to the DG group. The relative abundance of *Ruminobacter*, *Faecalibacterium*, *Bacteroides*, *Anaerovibrio*, *Alistipes,* and *Tyzzerella* had no significant difference displayed between the WG and DG groups. The relative abundance of gut bacterial communities at the genus level in the WG and DG groups is presented in (Table 4).

**Figure 5:**
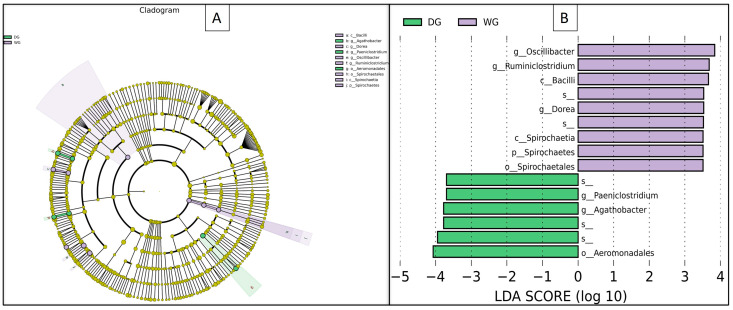
Cladogram showing differential bacterial taxa (**A**), Linear Discriminant Analysis (LDA) Effect Size (LEfSe) indicating biomarker taxa (**B**) in domestic grazing yaks (DG) and wild grazing yaks (WG).

Significant inter-individual variability was observed in the prokaryotic community composition at the phylum, family, and genus levels. To identify distinct microbial communities between the wild grazing (WG) and domestic grazing (DG) groups, Linear Discriminant Analysis Effect Size (LEfSe) was performed, including Linear Discriminant Analysis (LDA). The results revealed that the WG group was characterized by the presence of *Oscillibacter*, *Ruminiclostridium*, and Spirochaetes as biomarker taxa, whereas the DG group was distinguished by the presence of *Agathobacter*, *Aeromonadales*, and *Paeniclostridium* as biomarker taxa (Figure 5A,B).

### 3.7. Co-Occurrence Network Analysis

**Figure 6:**
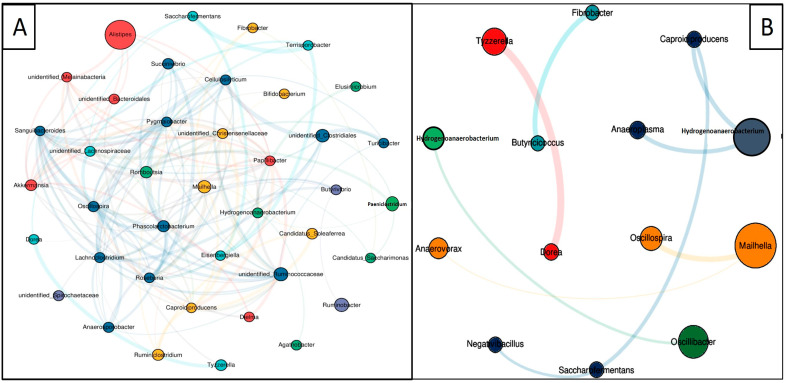
Co-occurrence network of significant bacterial genera. Potential mutualistic interactions of bacterial communities at genus level in (**A**) DG and (**B**) WG yak groups.

A co-occurrence network was constructed based on significant genera (*p* < 0.05), using Spearman’s test and an abundance of more than 0.001 and R-value (R < 0.04) to get insight into the potential mutualistic interaction of bacteria within each group (Figure 6A,B). The co-occurrence network of the DG group displayed that bacterial community complexity was at the peak in the DG group, as evident by the high number of nodes and edges. The total numbers of nodes and edges in the DG group were 40 and 146, and the numbers of positive and negative interactions were 86 and 60, respectively, while the co-occurrence network of the WG group displayed that the total numbers of nodes and edges in the WG group were 14 and 9, and the numbers of positive and negative interactions were 6 and 3, respectively (Table 5).

## 4. Discussion

Ruminants digest forage and other feedstuffs with the help of rumen microbiota to gain energy and other nutrients required for their growth and body maintenance [40]. The body’s immune system, physiological metabolism, nutritional absorption, growth, and development are all strongly correlated with intestinal flora. Numerous studies demonstrate that domestic yaks or domestic grazing yaks are inferior to wild yaks in many ways. To increase domestic yaks’ ability to produce, it is crucial to understand the structure of their intestinal bacterial flora. Previously, dozens of studies have explored patterns, associations, and the characterization of rumen microbial community structure in different environments and under different feeding systems to find similarities, variations, and associations between yaks and their rumen microbial communities. However, the gut bacterial community differences between wild grazing and domestic grazing yaks remain unclear. In the present study, we explored shifts in the gut bacterial communities of yaks influenced by the environment and herbage of pasture variations.

### 4.1. Forage Quality and Microbial Diversity

The ash, dry matter, and hemicelluloses contents of forages available to both the wild grazing and domestic grazing groups were the same. The free-range grazing pasture forages had higher levels of OM, poorly fermentable dietary fiber, including NDF ADF, and TC compared to the domestic grazing pasture forages. The variation in the chemical composition of forages is related to different factors such as plant species, diversity, ripeness, or growth stages [41], drying method, growth environment [42], and the kind of soil [43]. The bacterial diversity was higher during January, as the forage was dried and the NDF and ADF values were higher, as previously reported [44]. Alpha diversity analysis (using ACE, Chao1, and Shannon indices) demonstrated that the WG group had a more diverse fecal bacterial community compared to the DG group. This is consistent with existing research, which has found that high-fiber diets support more robust microbial populations, likely due to the superior ability of fiber fermentation to stimulate microbial growth compared to starch fermentation [45,46]. Fiber-based diets work well as prebiotics, as they contain more secondary plant compounds which assist in the enhancement of bacterial diversity [44,47].

### 4.2. Differences in Gut Mircobiota

In the present study, the WG yaks have different gut bacterial communities than the DG group, as they closely same the natural composition of forages, featuring similar levels of sugars, oligosaccharides, and peptic polysaccharides, which have been identified as crucial ingredients. Interestingly, the WG group exhibited an increase in the beta diversity of gut bacterial communities. Notably, the forage in both the WG and DG groups had similar hemicellulose content and higher digestible dry matter and crude protein levels, which may have contributed to the growth of the microbial community. Additionally, factors such as forage varieties, grazing patterns, environmental changes, and biomass may also play a role in shaping the diversity of the microbial communities in yaks [48]. 

### 4.3. Phylum-Level Bacterial Composition

At the phylum level, significant environmental, climatic, and some forage variations were observed in the rumen bacterial composition. Bacteroidetes and Firmicutes were the two dominant phyla in yak fecal samples, and many studies showed that Bacteroidetes and Firmicutes were the dominant phyla in yak rumen [49,50] and other ruminants [51,52]. The occurrence of these two dominant phyla in the rumen of yaks and many ruminants stipulates their biological and functional consequences. The main function of Bacteroidetes is the degradation of carbohydrates, proteins, and fats to produce energy, while Firmicutes are responsible for the generation of volatile fatty acids by the degradation of starch, cellulose, hemicelluloses, and oligosaccharide [53]. We observed a high abundance of Firmicutes in the fecal samples of the WG group, while the ratio of Bacteroidetes was higher in the DG group’s fecal sample, which indicates high fiber and low CP content in pasture herbage with a high abundance of Bacteroidetes, while high quality increased the abundance of Firmicutes in the rumen of yaks, as reported previously [54]. Both of these phyla displayed opposite trends as they changed location and forage nutrients; Firmicutes’ abundance was increased, while Bacteroidetes were decreased. 

These variations might be related to nutrient contents, location, and grazing pattern. Evaluation of climatic changes, forage nutrients, and altitude changes by differences in relative abundance at phylum level could better indicate phylum-specific functionalities of the bacterial community among the WG and DG groups. In this study, yaks had sparkling grass with greater CP and less crude fibers (NDF and ADF) during the growing period, while dry and snow-included grass was present in the course of the withering duration, which may have led to one-of-a-kind bacterial compositions at the phylum level. Changes in the relative abundances of Firmicutes and Bacteroidetes show the adaptation of the yak to climatic changes and forage nutrients, while an increase in Firmicutes and a decrease in Bacteroidetes was reported in yaks, which is consistent with our results [28]. Other studies also concluded that variations in diet, climate, and grazing pattern are also responsible for the Bacteroidetes’ or Firmicutes’ dominancy [24]. However, in a firm geographical area, diet composition and host species had little influence on the prevailing point of these two bacterial phyla [55].

Moreover, the Firmicutes-to-Bacteroidetes proportion plays a vital role in assessing the consequences of gut bacterial effect on host energy provisions [56]. In the present study, the Firmicutes-to-Bacteroidetes ratio in the WG group was noticeably higher than in the DG group. Therefore, yaks fed with no time restriction in natural grazing environments obtained more energy at ease, which is considered important for body metabolism. According to previous findings, the Firmicutes-to-Bacteroidetes ratio is associated with roughage proportion and milk-fat yield [57]. Some studies have reported that the ratio of Firmicutes were greater in Qinghai–Tibetan Plateau (QTP) sheep than plain land sheep and goats. The role of Gram-positive bacteria is very important in the digestion of existing grasses at the QTP [58]. These findings are consistent with the present study, as the location and grazing pattern changes might have preceded the ratio of Firmicutes in the WG and DG groups. Favorable conditions for Firmicutes’ proliferation are dependent on environmental changes, forage worth, and varieties of available forage. In this study, a higher abundance of Firmicutes is related to different factors, such as location [58], age [59], and diet [60]. According to previous findings, Firmicutes’ abundance is directly proportional to an increase in starch and fat-rich high-energy diets [61]. Consistent with previous research, the dominant bacterial phyla in the rumen of grazing yaks in the QTP were found to be Firmicutes, Bacteroidetes, and Proteobacteria [55,58,62]. Our study’s results are in agreement with previous findings, showing that Firmicutes, Bacteroidetes, and Proteobacteria were the dominant phyla in both the WG and DG groups. We also found that their abundances were influenced by dietary patterns and environmental factors, as previously reported [58,60].

### 4.4. Family-Level Bacterial Composition

The predominant families in the WG and DG groups were *Ruminococcaceae*, *Rikenellaceae*, *Lachnospiraceae*, *Bacteroidaceae*, *Prevotellaceae*, and *Succinivibrionaceae*, with *Ruminococcaceae* being more abundant in the WG group than the DG group. These bacterial families are crucial for fiber and starch degradation, as well as enhanced fiber digestibility [63]. In the grazing pasture, the available high-quality forages and a sufficient amount of nutrients enhanced the quantity of fiber-degrading bacteria such as *Ruminococcaceae* and *Rikenellaceae*. Similar studies have reported that *Ruminococcaceae* is responsible for the degradation of proteins [64]. *Lachnospiraceae* play a crucial role in the growth stimulation of fibrolytic bacteria found in Holstein cows’ rumens, as reported previously [65,66]. This study also reported that *Prevotellaceae* lowers the nitrogen thrashing, produces acetate as the fermentation final product, and improves forage consumption [67]. In the present study, *Rikenellaceae, Lachnospiraceae,* and *Succinivibrionaceae* in the DG group were higher than in the WG group. This study reported that *Prevotellaceae* is a dominant bacterium of the saccharolytic group in the rumen and is also important for its protein binding capacity and also digestion of several carbohydrate substrates [68]. The *Prevotellaceae* in feces of the yaks with high abundance indicated high carbohydrate degradation capability. Previous studies have reported that *Ruminococcaceae, Rikenellaceae*, and *Prevotellaceae* are considered as the most important families for forage degradation in the rumens of ruminants until these bacteria tightly stick to forage grass after staying in the rumen [69,70]. These findings are consistent with the present study; a higher ratio of *Ruminococcaceae* and *Rikenellaceae* in the WG and DG groups, respectively, are anticipated to improve fiber degradation.

### 4.5. Genus-Level Bacterial Composition

At the genus level, among the dominant genera, *Bacteroides, Alistipes*, *Alloprevotella*, *Ruminobacter*, *Succinivibrio*, and *Faecalibacterium* were the most dominant genera in the WG and DG groups. In the rumens of ruminants, *Ruminobacter* is important for degrading starch into acetate and propionate [71]. In the rumen of Holstein cows, the abundance of this genus has been reported to be linked with concentrate diet [72], which is in concordance with our results. *Succinivibrio* is a starch-degrading bacterial genus that makes primarily acetate and succinate. Prevotella, belonging to *Alloprevotella,* has been characterized by huge genetic variance and possesses efficient adaptability [73], which is important for preliminary dietary protein breakdown [74], starch degradation, proficient utilization of hemicelluloses [75], and peptide metabolism [76]. *Alloprevotella* has the same work flexibility as Prevotella. *Alloprevotella, Ruminobacter* and *Succinivibrio,* all three dominant genera, boost fiber degradation. The most dominant and important bacterial genus, *Bacteroides,* in the intestinal microbial population of diarrheal yaks, can sop up the nutrients and make short-chain fatty acids (SCFAs) [77], helping in recovering and enhancing the maturation of epithelial cells associated with the fat’s metabolism. Moreover, an *Alistipes* commensal bacterial genus has the maximum quantity of putrefaction. Putrefaction is a process in which the undigested protein fermentation occurs in the gastrointestinal tract by the gut microbiota and produces harmful metabolites [78]. Our study showed that the *Alistipes* genus is less dominant, having no significant differences between two groups. *Faecalibacterium* produce butyrate to support the safeguarding of intestinal mucosa [79] and shield them from inflammation [80]. 

Many proteins are encoded by *Bacteroides* and also have the ability to carry out complex carbohydrates and degrade them [68]. *Bacteroides* and *Faecalibacterium* help in making SCFAs, which leads in balancing the gut microbe’s structure in yaks and also helps in maintaining the intestinal epithelium. In the current study, *Alloprevotella* and *Succinivibrio* were more abundant in the DG group than the WG group. *Succinivibrio* promotes the production of SCFAs and microbial growth. As a member of the *Alloprevotella* genus, Prevotella has the capacity to break down dietary fibers from plant cell walls, resulting in the production of a significant amount of SCFAs [81]. Prevotella that metabolize dietary fibers, *Alloprevotella,* also boost its metabolism. These findings might be endorsed by the nutrient composition of forages and grazing patterns, as higher OM contents increase the growth of these fibrolytic bacteria. 

Overall, these variations make known important information related to the nutrient composition of forages and the grazing patterns of different yaks. Forage quality, quantity, varieties, feeding pattern, and environmental effects could be associated with bacterial composition, diversity, and functions of the bacterial community in yak feces. Sufficient amounts of high-quality forage, nutrients, and forage quantity in grazing pastures for yaks improved the gut bacterial diversity.

## 5. Conclusions

This study investigated the composition and diversity of the fecal bacterial communities in WG and DG groups, revealing that feeding patterns influenced the structure and variation of the fecal bacterial community. Specifically, Firmicutes were more abundant in the WG group, which grazed on natural pastures, than in the DG group. In this study, we also found that changes in the fecal bacterial communities of yaks may be related to their health and external environmental adaptation. The WG group grazing on natural pastures favored the fiber-degradation bacteria (related to *Ruminococcaceae*), while the DG group improved the abundance of protein and carbohydrate-degrading (*Prevotellaceae*). Studying the effects of these factors contributes novel insights into the current understanding of the fecal bacterial communities of yaks to help us in our future understanding of the relation between microbes and the host, provides suggestions for better background, and also provides new approaches to prebiotics and microbiological agents.

## Figures and Tables

**Table 1:** The chemical composition of forages in domestic grazing yaks (DG) and wild grazing yaks (WG), natural grazing pasture.

Parameters	Forage in DG	Forage in WG	SEM	*p*-Value
Dry matter	97.00%	96.96%	0.047	0.652
Organic matter	92.58%	92.63%	0.035	0.716
Ash	7.30%	7.29%	0.009	0.345
Crude protein	15.10%	7.30%	5.025	0.001
Neutral detergent fiber	53.78%	57.68%	2.757	0.001
Acid detergent fiber	27.8%	30.81%	2.133	0.012
Ether extract	1.37%	1.5%	0.091	0.029
Total carbohydrates	76.53%	77.43%	0.636	0.095
Non-fiber carbohydrates	22.76%	22.1%	0.471	0.034
Hemicellulose	26.13%	26.33%	0.141	0.467

**Table 2:** The relative abundance of gut bacterial communities at the phylum level in the WG and DG groups.

Taxonomy	DG	WG	SEM	*p*-Value
Bacteroidetes	0.91 ^b^	1.08 ^a^	0.119	0.052
Firmicutes	1.12 ^a^	0.89 ^b^	0.162	0.024
Proteobacteria	0.93	1.06	0.088	0.505
Elusimicrobia	1.07 ^a^	0.93 ^b^	0.098	0.032
Fusobacteria	1.18 ^a^	0.84 ^b^	0.246	0.001
Kiritimatiellaeota	1.08 ^a^	0.92 ^b^	0.114	0.042
Lentisphaerae	1.15	0.86	0.198	0.0002
Spirochaetes	0.81	1.22	0.293	0.108
Fibrobacteres	0.86 ^b^	1.15 ^a^	0.202	0.059
Tenericutes	0.93	1.07	0.098	0.297
Verrucomicrobia	1.09	0.91	0.127	0.096
Melainabacteria	1.06	0.94	0.083	0.127

Values were log transformed. Means in a row with different small-letter superscripts differ significantly (*p* < 0.05); same-letter superscripts present no difference (*p* > 0.05).

**Table 3:** The relative abundance of gut bacterial communities at the family level in the WG and DG groups.

Taxonomy	DG	WG	SEM	*p*-Value
Ruminococcaceae	1.22 ^a^	0.81 ^b^	0.286	0.0001
Succinivibrionaceae	0.89	1.11	0.160	0.562
Prevotellaceae	0.85 ^b^	1.16 ^a^	0.219	0.0003
Lachnospiraceae	0.82	1.21	0.271	7.889
Rikenellaceae	0.97	1.03	0.042	0.372
Bacteroidaceae	0.96	1.04	0.056	0.161
Muribaculaceae	0.84 ^b^	1.18 ^a^	0.244	0.043

Values were log transformed. Means in a row with different small-letter superscripts differ significantly (*p* < 0.05); same-letter superscripts present no difference (*p* > 0.05).

**Table 4:** The relative abundance of gut bacterial communities at the genus level in the WG and DG groups.

Taxonomy	DG	WG	SEM	*p*-Value
Succinivibrio	0.78	1.26	0.340	0.060
Ruminobacter	0.92	1.07	0.103	0.742
Alloprevotella	0.89	1.11	0.160	0.068
Faecalibacterium	0.90	1.10	0.144	0.252
Bacteroides	0.96	1.04	0.056	0.161
Anaerovibrio	0.89	1.12	0.162	0.448
Alistipes	1.02	0.97	0.030	0.487
Mailhella	1.07 ^a^	0.93 ^b^	0.099	0.018
Tyzzerella	1.03	0.96	0.047	0.105

Values were log transformed. Means in a row with different small-letter superscripts differ significantly (*p* < 0.05); same-letter superscripts present no difference (*p* > 0.05).

**Table 5:** Topological features of co-occurrence network analysis among the DG and WG groups at the genera level.

S. No	Network Attributes	DG	WG
1	Nodes	40	14
2	Edges	146	9
3	Average degree	7.3	1.285714286
4	Average path length	2.457692308	1.6875
5	Network diameter	4	3
6	Clustering coefficient	0.577793009	0
7	Density	0.187179487	0.098901099
8	Heterogeneity	0.72033543	0.364627846
9	Centralization	0.223076923	0.054945055
10	Positive correlation	58.90%	66.66%
11	Negative correlation	41.10%	33.34%

## Data Availability

The datasets presented in this study can be found in online repositories. The names of the repository/repositories and accession numbers can be found at https://www.ncbi.nlm.nih.gov/sra/PRJNA981343 (accessed on 1 August 2024).
